# High-pressure studies in the supercooled and glassy state of the strongly associated active pharmaceutical ingredient—ticagrelor

**DOI:** 10.1038/s41598-023-35772-7

**Published:** 2023-06-01

**Authors:** Paulina Jesionek, Dawid Heczko, Barbara Hachuła, Kamil Kamiński, Ewa Kamińska

**Affiliations:** 1 Institute of Chemistry, Faculty of Science and Technology, University of Silesia in Katowice, Szkolna 9, 40-007 Katowice, Poland; 2 Department of Pharmacognosy and Phytochemistry, Faculty of Pharmaceutical Sciences in Sosnowiec, Medical University of Silesia in Katowice, Jagiellonska 4, 41-200 Sosnowiec, Poland; 3 Department of Statistics, Department of Instrumental Analysis, Faculty of Pharmaceutical Sciences in Sosnowiec, Medical University of Silesia in Katowice, Ostrogorska 30, 41-200 Sosnowiec, Poland; 4 Institute of Physics, Faculty of Science and Technology, University of Silesia in Katowice, 75 Pulku Piechoty 1, 41-500 Chorzow, Poland

**Keywords:** Glasses, Physical chemistry

## Abstract

In this paper, the molecular dynamics at different thermodynamic conditions of hydrogen-bonded (H-bonded) active pharmaceutical ingredient—ticagrelor (TICA) have been investigated. Extensive high-pressure (HP) dielectric studies revealed surprising high sensitivity of the structural (*α*)-relaxation to compression. They also showed that unexpectedly the shape of the *α*-peak remains invariable at various temperature (*T*) and pressure (*p*) conditions at constant *α*-relaxation time. Further infrared measurements on the ordinary and pressure densified glasses of the examined compound indicated that the hydrogen-bonding pattern in TICA is unchanged by the applied experimental conditions. Such behavior was in contrast to that observed recently for ritonavir (where the organization of hydrogen bonds varied at high *p*) and explained the lack of changes in the width of *α*-dispersion with compression. Moreover, HP dielectric measurements performed in the glassy state of TICA revealed the high sensitivity of the slow secondary (*β*)-relaxation (Johari–Goldstein type) to pressure and fulfillment of the isochronal superpositioning of *α*- and JG-*β*-relaxation times. Additionally, it was found that the activation entropy for the *β*-process, estimated from the Eyring equation (a high positive value at 0.1 MPa) slightly increases with compression. We suggested that the reason for that are probably small conformational variations of TICA molecules at elevated *p*.

## Introduction

Glass-forming materials have been the subject of intensive research in recent decades^1–4^. A special attention is paid to the molecular understanding of the vitrification process and phenomena occurring in the vicinity of the glass transition. It is worth mentioning that intensive studies carried out on various types of glass-formers, i.e., polymers, low-molecular-weight (LMW) organic and inorganic compounds, ionic liquids, etc.^5–9^, have revealed two characteristic features of these systems. The first one is the continuous and rapid increase of the structural relaxation time (*τ*_*α*_) from values of the order of picoseconds (typical for liquids) up to hundreds of seconds near the glass transition temperature (*T*_g_), while the second one is a non-exponential (in a time-domain) or non-Debye (in a frequency domain) character of the relaxation function. It should be mentioned that according to the literature data, the width of the *α*-dispersion close to the *T*_*g*_ (reflected in the stretched exponent of the Kohlrausch–Williams–Watts (KWW) function, *β*_KWW_) is considered as either an inherent feature of supercooled liquids or the measure of the heterogeneity in the investigated systems^4^. Thus, with regard to this claim, the response relaxation function of the materials in the vicinity of the *T*_g_ consists of the series of Debye relaxations characterized by different relaxation rates. Recently, an important step forward in understanding the asymmetric shape of the structural (*α*)-process in supercooled liquids has been made. In ref.^10^, it was shown that the polarity of the molecules is a key parameter that may have a strong impact on the breadth of the *α*-mode. The authors analyzed over 180 van der Waals materials and concluded that along with the increasing dipole moment/ dielectric relaxation strength, the narrowing of the *α*-loss peak is noted. What is more, the observed phenomenon was correlated with the anharmonicity of the potential well describing intermolecular interactions in examined systems. Having that in mind, it would be valuable to confront this supposition with high-pressure investigations that induce strong densification of the sample and consequently change the shape as well as the depth of the potential well. Interestingly, the vast majority of the data collected for the purely van der Waals systems indicated that the width of *α*-dispersion is solely governed by the relaxation times and remains unchanged regardless of different thermodynamic conditions^4,11–16^. This means the fulfillment of the very important rule called temperature–pressure superpositioning (TPS)^11^. However, there are some substances having silyl/acetyl units in their chemical structures, for which the above rule is not satisfied. Herein, one can briefly mention LMW non-associated compounds, such as tris(dimethylsiloxy)phenylsilane (TDMSPS)^17^, octa(trimethyl)silyl and octaacetyl trehalose (silTRE and acTRE)^18^, where the narrowing of the *α*-loss peak under elevated pressure is observed. Another family of compounds where the TPS law is not obeyed are strongly associating liquids forming extensive H-bonded networks, i.e., *m*-fluoroaniline^19^, polyalcohols (glycerol, xylitol, threitol)^20^, di-, tri-propylene glycols^21^. Importantly, in these materials, the broadening of the *α*-dispersion with compression is noted. Such a finding was explained by the researchers as resulting from the variations in the strength and population of hydrogen bonds (H-bonds) as well as density fluctuations at extreme thermodynamic conditions, thereby causing the changes in the physical structures^11^. However, it turns out that there are a few H-bonded compounds where the influence of *T* and *p* on the width of the structural relaxation peak is not observed^22–25^. Thus, the question arises, why in some compounds forming H-bonds, there is a pronounced impact of thermodynamic conditions on the shape of the *α*-dispersion, while in others—it is not. To gain some insight into this problem, we decided to study the behavior of pressure densified glasses made of two active pharmaceutical ingredients (APIs): ticagrelor and ritonavir (for comparison). As can be shown in this paper, the change in the population of H-bonds has, in fact, an impact on the variation in the shape of the structural process at various *T* and *p* conditions.

## Results and discussion

### Differential scanning calorimetry (DSC) data

**Figure 1:**
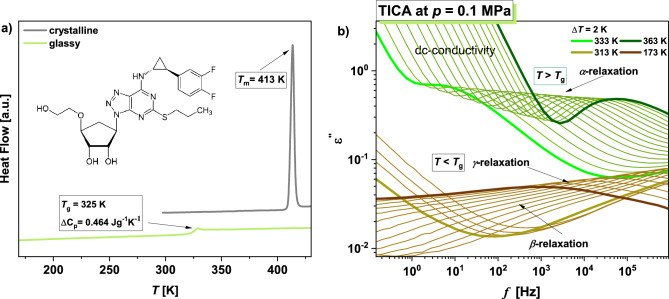
DSC thermogram obtained during heating of the crystalline and vitrified TICA at the rate of 10 K/min (**a**). In the inset, the chemical structure of the examined API is presented. Panel (**b**) presents dielectric loss spectra measured for TICA at ambient pressure in the indicated temperature (*T*) ranges.

At first, we have carried out calorimetric measurements to entirely characterize the thermal properties of TICA. In panel (a) of Fig. 1, DSC curves recorded on heating (10 K/min) the crystalline and glassy TICA are presented. As can be seen, there is one endothermic event in the thermogram of the crystalline sample at *T*_*m*_ = 413 K, corresponding to the melting process. Further cooling, followed by heating of the vitrified TICA, reveals the presence of a well-visible heat capacity jump at *T*_g_ = 325 K $(\Delta \mathrm{C_p}$ = 0.464 Jg^−1^ K^−1^), associated with the glass transition phenomenon. It should be mentioned that similar values of $\Delta$ C_p_ parameter (i.e., change in heat capacity at *T*_g_) have been reported for other H-bonded APIs, such as bisoprolol ($\Delta \mathrm{C_p}$ = 0.510 Jg^−1^ K^−1^)^26^, posaconazole ($\Delta \mathrm{C_p}$ = 0.480 Jg^−1^ K^−1^)^27^, ezetimibe ($\Delta \mathrm{C_p}$ = 0.470 Jg^−1^ K^−1^)^28^, indapamide ($\Delta \mathrm{C_p}$ = 0.490 Jg^−1^ K^−1^)^28^, indomethacin ($\Delta \mathrm{C_{p}}$ = 0.466 Jg^−1^ K^−1^)^29^ or valsartan ($\Delta \mathrm{C_p}$ = 0.420 Jg^−1^ K^−1^)^30^. Note that they exceeded those determined for, e.g., terconazole, ketoconazole, and itraconazole ($\Delta \mathrm{C_p}$ = 0.391, 0.366, 0.260 Jg^−1^ K^−1^, respectively)^27^, as well as for griseofulvin ($\Delta \mathrm{C_p}$ = 0.272 Jg^−1^ K^−1^)^31^, which are typical van der Waals systems.

### Broadband dielectric spectroscopy (BDS) data

In the next step, we have performed molecular dynamics BDS studies on the supercooled/vitrified (ordinary glass, OG) of TICA at ambient pressure, in a wide range of *T*, both above and below the glass transition temperature (*T*_g_). Dielectric loss spectra obtained from these investigations are illustrated in panel (b) of Fig. 1. In the supercooled liquid phase (*T* > *T*_g_) of the examined compound, one can distinguish two characteristic processes in the spectra. The first one is the dc-conductivity, connected to the charge transport of ionic impurities and also, due to the presence of many hydroxyl groups in the TICA structure, to the proton hopping. Consequently, the measured conductivity is a result of both types of effects. In turn, the second one, located at higher frequencies (*f*), is the structural (*α*)-relaxation associated with the cooperative motions of all molecules and responsible for the glass transition. As illustrated, both processes shift towards lower *f* with decreasing *T*. In turn, in the glassy state (*T* < *T*_g_), two secondary relaxations with small amplitude (labeled as *β* and $\gamma$) are observed in dielectric loss spectra.

**Figure 2:**
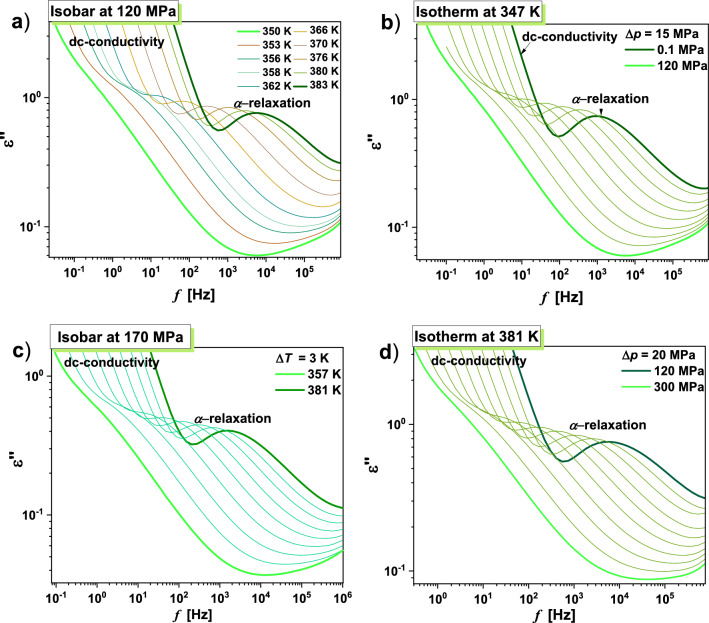
Representative dielectric loss spectra measured for TICA at isobaric (panels (**a**) and (**c**)) and isothermal (panels (**b**) and (**d**)) conditions.

**Figure 3:**
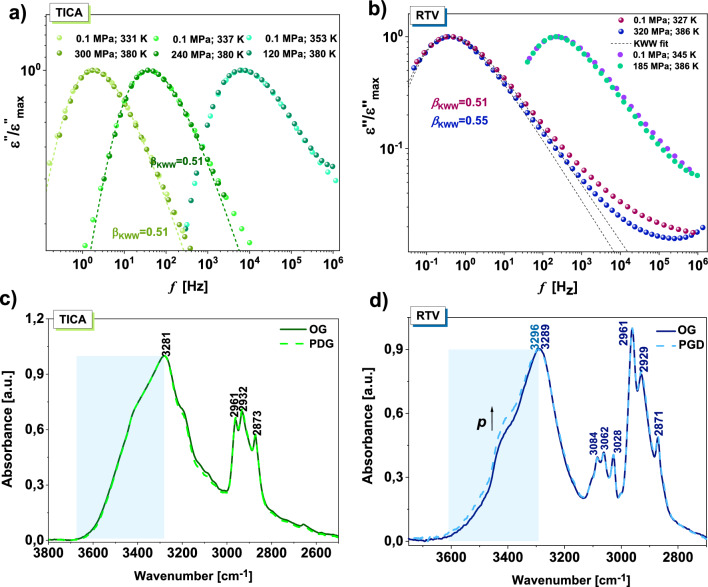
A superpositioning of TICA (**a**) and RTV^32^ (**b**) spectra collected at three (two) different thermodynamic conditions close and far above the *T*_g_. The spectra were normalized with respect to the maximum of dielectric loss (*ε*”_max_). The dashed lines represent KWW fits. Panels (**c**) and (**d**) present FTIR spectra measured at 298 K for the OG as well as PDG of TICA and RTV^32^, respectively.

Having ambient pressure dielectric spectra of TICA described qualitatively, we shift our attention to the behavior of this API at varying thermodynamic conditions. The results of isobaric and isothermal experiments performed at indicated thermodynamic conditions are presented in Fig. 2. Panels (a) and (c) of this figure demonstrate representative dielectric loss spectra measured at constant *p* and various *T* > *T*_g_, while panels (b) and (d) present the analogical data obtained at constant *T* and different *p* < *p*_*g*_ (where *p*_*g*_ means the glass transition pressure). As in the case of ambient pressure data, besides the dc-conductivity, a single structural (*α*)-relaxation peak is apparent in the loss spectra of the studied API at elevated compression. Its maximum shifts toward lower *f* with decreasing *T* or increasing *p*. The subsequent stage of our investigations was to compare the normalized dielectric spectra collected for TICA at various thermodynamic conditions in the vicinity of the *T*_g_, see panel (a) of Fig. 3. It should be mentioned that the dc-conductivity part was subtracted from the spectra to better visualize the shape of the *α*-relaxation peak.

As illustrated, the width of the *α*-dispersion remains unchanged regardless of combinations of *T* and *p*, which indicates the fulfillment of the TPS rule^11^. This is confirmed by the outcomes of fitting the normalized spectra to the one-sided Fourier transform of the KWW function (dashed lines)^33,34^:

1 $${\Phi }_{KWW}\left(t\right)=\mathrm{exp}[-(t/{{\tau }_{\alpha })}^{{\beta }_{KWW}}].$$

The obtained value of the stretched exponent (${\beta }_{KWW})$ was the same, equal to 0.51. This result is very surprising considering the presence of three O–H and one N–H groups in the TICA molecule (and hence the probability of formation of strong H-bonds/extensive H-bonded networks). In such a situation, it is highly expected that the application of high *T* and *p* should affect the population and strength of these specific interactions, which further should be reflected in the variation in the dielectric response function, as it was previously reported for many H-bonded systems^19–21,35^. Therefore, a question arises, is there a direct connection between the H-bonding pattern and the shape of the structural process, or maybe alternatively, pressure and temperature effects compensate (consequently, there is a marginal impact of thermodynamic conditions applied in our experiments on the strength of H-bonds)? To verify which of these theses is the true one, complementary Fourier-transform infrared spectroscopy (FTIR) investigations at high *p* must be done. Unfortunately, at the moment, we do not have access to such equipment. To overcome these limitations, we decided to carry out FTIR studies on the pressure densified glass (PDG) of TICA. Herein, one can mention that the above approach allows us to freeze the higher density and structure of the compressed sample and potentially obtain a valuable insight into specific interactions at high pressure^36^. In order to produce the PDG, as a first, the sample of TICA was compressed up to *p* = 400 MPa at *T* = 385 K. Next, it was cooled to *T* = 283 K (much below the *T*_g_), and then the pressure was released. Just after decompression, the PDG of API was measured by means of FTIR spectroscopy to avoid any significant effect of equilibration/ physical aging.

In panel (c) of Fig. 3, the infrared spectra measured at room temperature for the PDG, as well as the OG of TICA are presented. As illustrated, the spectral profile corresponding to the stretching vibrations of the O–H and N–H groups in the densified glass (PDG) remains unchanged with respect to the OG. This indicates a stable distribution of H-bonds in both samples and thus confirms that, most likely, there is a compensating effect of densification and higher thermal energy on the strength of this kind of interactions at high *p*. As a consequence, at isochronal conditions (*τ*_*α*_ = const.), the shape of the structural process remains unchanged. Herein, one can refer to our recent paper, where a similar methodology has been applied to understand a peculiar narrowing of the width of the structural process in ritonavir (RTV) at high compression^32^. In panel (d) in Fig. 3, infrared spectra of OG and PDG of this active substance are shown. As can be seen, the spectral profile of the O–H and N–H stretching vibration bands varies significantly due to compression. The observed changes indicate the increasing population of the weaker H-bonds in the PDG. This phenomenon was reflected in the variation of the shape of the structural process (its narrowing) at constant structural relaxation time (see Fig. 3b). Both mentioned examples (TICA and RTV) suggest that, in fact, there is a relationship between the shape of the structural process and the H-bonding pattern at high pressure.

**Figure 4:**
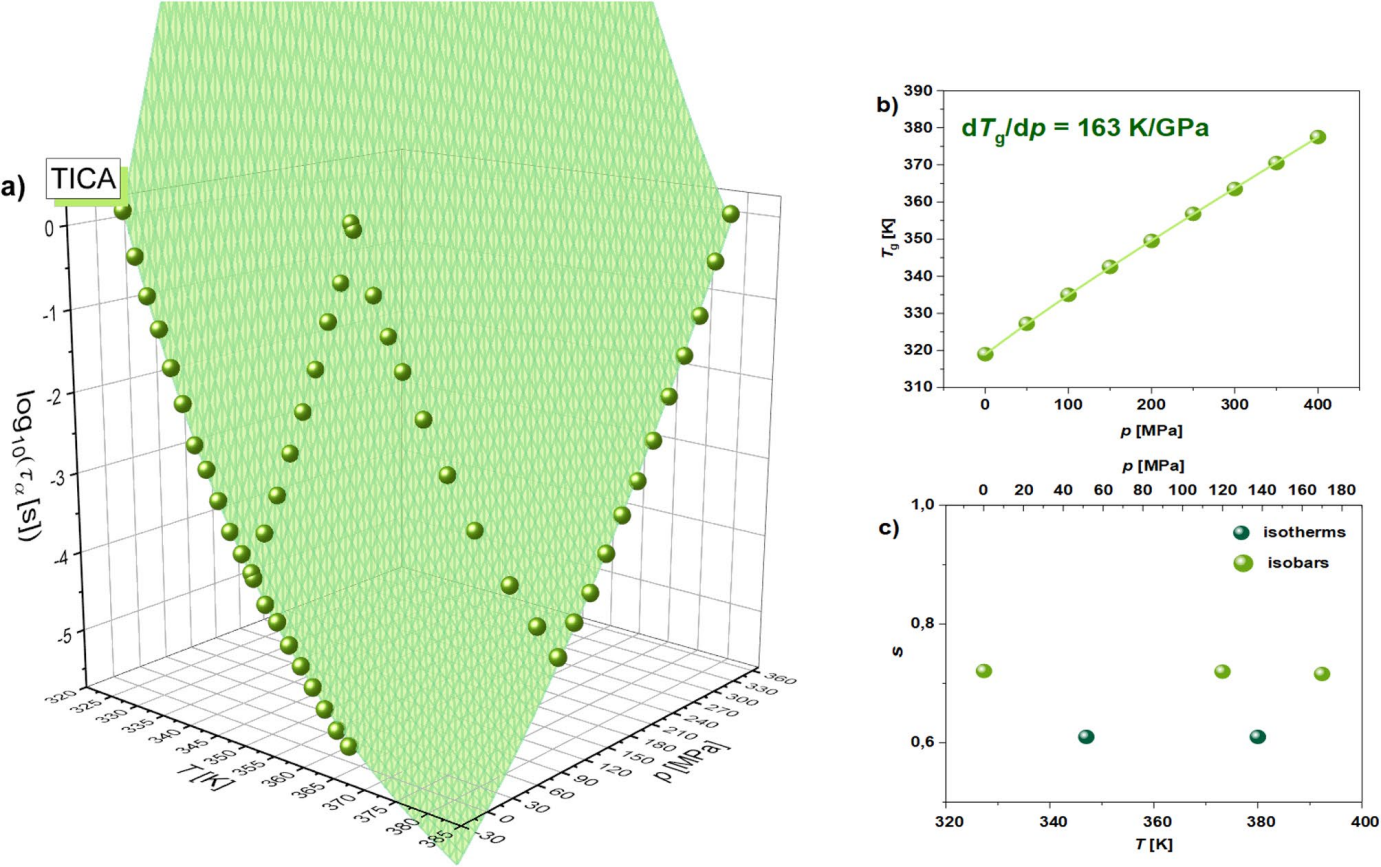
Structural (*α*)-relaxation times plotted as a function of *T* and *p* for TICA (**a**). The green area represents surface fits to equation S1. *T*_g_ versus *p* plot (**b**) and the dependence of the parameter $s$ from Eq. (5) as a function of *T* and *p* (**c**).

Subsequently, we performed a comprehensive analysis of dielectric spectra determined for TICA from isobaric to isothermal measurements. First of all, the loss spectra collected at various thermodynamic conditions in the liquid/supercooled liquid state were fitted to the Havriliak–Negami (HN) function with the conductivity term (Eq. 2)^37^:

2 $$\varepsilon (\varpi )"=\frac{{\sigma }_{dc}}{{\varepsilon }_{0}\varpi }+{\varepsilon }_{\infty }+\frac{\Delta \varepsilon }{{\left[1+{\left(\mathrm{i}\overline{\upomega }{\tau }_{HN}\right)}^{\alpha }\right]}^{\gamma }},$$

where ${\sigma }_{dc}$ is the dc-conductivity,* ε*_0_ is the vacuum permittivity, $\overline{\omega }$ is an angular frequency ($\overline{\omega }$ = 2π*f*), ${\varepsilon }_{\infty }$ is the high-frequency limit permittivity, Δ*ε* is the dielectric relaxation strength, ${\tau }_{HN}$ is the HN relaxation time, *α* and *γ* are the shape parameters representing the breadth and asymmetry of given relaxation peaks. Then, in panel (a) of Fig. 4, we plotted the obtained *α*-relaxation times $, {\tau }_{\alpha }$ (firstly recalculated from ${\tau }_{HN}$ using the formula given in ref. ^38^) versus temperature and pressure. Next, these ${\tau }_{\alpha }$ (*T*, *p*) dependencies, which created two-dimensional surfaces within the considered *T* and *p* range, were analyzed using the modified Avramov expression^39^ given in the Supplementary Information, SI (equation S1). Note that the accuracy of the Avramov fits is illustrated in Fig. S1 presenting the separate isobars and isotherms determined for TICA together with the fits using equation S1.

Afterwards, the values of *T*_g_ obtained from the following formula proposed by Avramov^40^:

3 $$T_{g} \left( p \right) = T_{g} \left( {p_{0} } \right)\left( {1 + \frac{p}{\Pi }} \right)^{{{\beta \mathord{\left/ {\vphantom {\beta {\alpha _{0} \left( {1 - \frac{C}{{C_{{p_{0} }} }}\left( {{\text{ln}}\left( {1 + \frac{p}{\Pi }} \right)} \right)} \right)}}} \right. \kern-\nulldelimiterspace} {\alpha _{0} \left( {1 - \frac{C}{{C_{{p_{0} }} }}\left( {{\text{ln}}\left( {1 + \frac{p}{\Pi }} \right)} \right)} \right)}}}} ,$$

with the same parameters ($\Pi$, $\beta$, ${\alpha }_{0}$, and ${C}_{{p}_{0}}$) as those in equation S1 were plotted versus pressure (see Fig. 4b). From this dependence, we estimated the pressure coefficient of the glass transition temperature (d*T*_g_/d*p*)—a parameter reflecting the sensitivity of the structural dynamics to compression. Its value in the limit of ambient pressure (= 163 K/GPa) indicates a relatively strong influence of pressure on the *α*-relaxation in TICA. Note that a similar but quite higher value of d*T*_g_/d*p* has been reported for RTV (~ 200 K/GPa)^32^ and some weakly associated pharmaceuticals, such as droperidol^24^, ibuprofen^41^, or curcumin^42^. Importantly, the values in the range 160–220 K/GPa significantly exceed those determined for the other LMW compounds (d*T*_g_/d*p* < 70 K/GPa)^20,22^, which, similarly to TICA, are strongly H-bonded systems.

Additionally, we have decided to check whether for TICA there is a correlation between the dc-conductivity, ${\sigma }_{dc}$ (associated with the charge transport and proton hopping) and *α*-relaxation times, ${\tau }_{\alpha }$ (reorientation dynamics) according to the Debye–Stokes–Einstein (DSE) law:

4 $${\sigma }_{dc}{\tau }_{\alpha }=const,$$

Due to the fact that the above relation is very often not fulfilled, another form of Eq. (4), which is called the fractional DSE law (FDSE), has been applied^43^:

5 $${\sigma }_{dc}^{s}{\tau }_{\alpha }=const \left(0<s<1\right),$$

where $s$ is the fractional exponent describing the slope of dependency ${\sigma }_{dc}$ versus ${\tau }_{\alpha }$; see Fig. S2a,b in the SI). It should be mentioned that when $s$ ~1, there is a coupling between ${\sigma }_{dc}$ and ${\tau }_{\alpha }$, while its lowering (to values closer to 0) suggests the increase of decoupling between both quantities. In panel (c) of Fig. 4, we plotted the values of $s$ (determined from the linear fitting of the isobaric and isothermal data shown in Fig. S2 in the SI) versus pressure and temperature. As can be seen, the fractional exponent practically does not vary with increasing *p* and* T*. This experimental observation might be related to the unchanged H-bonding pattern at higher compression as deduced from FTIR investigations on the PDG of TICA. As a consequence, proton hopping—an important factor contributing to the overall dc-conductivity of the sample, is most likely not affected by the thermodynamic conditions applied in our experiments. Nevertheless, it should be stressed that there is a clear decoupling between ${\sigma }_{dc}$ and structural dynamics in the examined sample at each investigated isotherm and isobar ($s \approx \mathrm{0,6}-\mathrm{0,7}$). It is an expected result since we deal with the H-bonded liquid, where proton hopping strongly contributes to the measured dc-conductivity. It is worth noting that a pronounced decoupling between ${\sigma }_{dc}$ and ${\tau }_{\alpha }$ has been also reported in the case of another associated API, curcumin ($s$ =0.87 at *p* = 0.1 MPa)^42^. However, for this substance, the fractional exponent, $s,$ changed in a slightly wider range, from 0.87 to 0.73 and from 0.7 to 0.63 with increasing *p* and *T*, respectively. A similar scenario has been also found for the anhydrosaccharide—1,6-anhydro-D-glucose^22^. Herein, the value of $s$ varied from 0.95 (*p* = 0.1 MPa) to 0.68 at *p* = 255 MPa.

**Figure 5:**
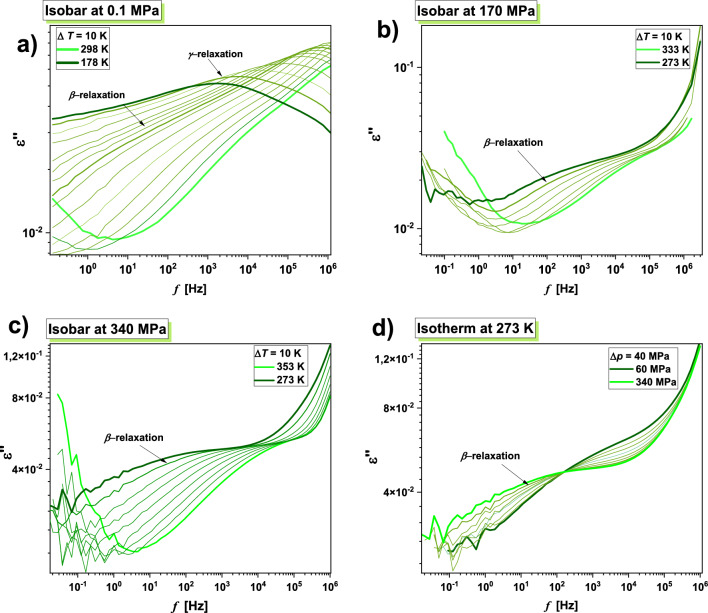
Dielectric spectra measured for TICA at 0.1 MPa (**a**), 170 MPa (**b**) and 340 MPa (**c**) and indicated *T* < *T*_g_. Panel (**d**) represents isothermal data (*T* = 273 K) collected in the *p* range 60–340 MPa.

In the subsequent stage, we focused on characterizing the influence of high compression on the molecular dynamics of TICA in the glassy state. Representative dielectric loss spectra collected at isobaric conditions (i.e., at *p* = 0.1, 170 and 340 MPa), and indicated temperatures below *T*_g_, as well as isothermal conditions (*T* = 273 K) and *p* > *p*_g_ are illustrated in Fig. 5. As can be seen, in contrast to ambient *p* dielectric data (panel a), which revealed the presence of two secondary modes labeled as *β* and *γ,* at elevated *p*, only one well-resolved *β*-relaxation peak can be observed in the presented spectra. The maximum of *γ*-process is out of the experimental frequency window, which suggests its insensitivity to density changes.

**Figure 6:**
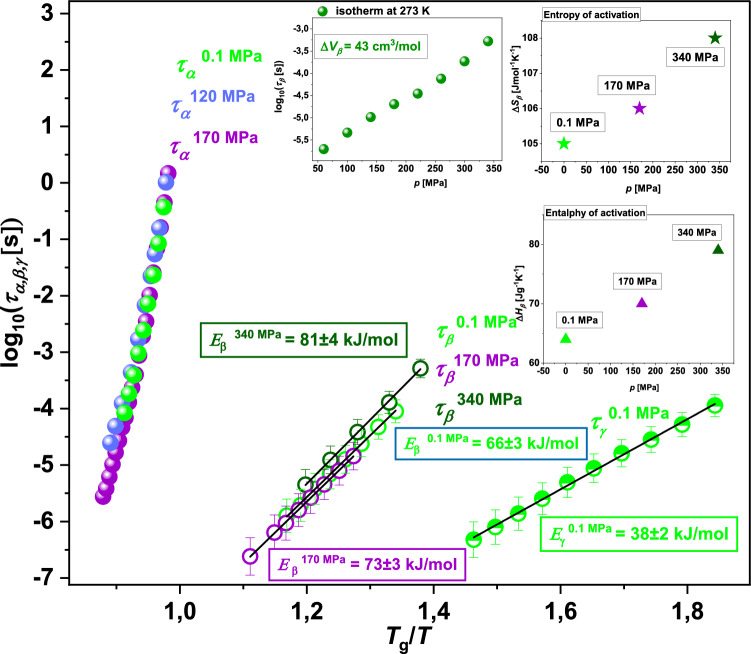
Dependences of relaxation times *τ*_*α*_, *τ*_*β*_, *τ*_*γ*_ versus *T*_g_/*T* for TICA. The solid lines are Arrhenius fits (Eq. 6). In the insets, log_10_*τ*_*β*_ plotted versus *p*, as well as $\Delta {S}_{\beta }$ and $\Delta {H}_{\beta }$ calculated at ambient and elevated pressure conditions from the Eyring equation (Eq. 8) are presented.

In order to obtain relaxation times of secondary (*β*)-process at various thermodynamic conditions (as well as *τ*_*γ*_ at *p* = 0.1 MPa), the loss spectra presented in Fig. 5 were fitted to the HN formula, Eq. (2) (actually, we applied the superposition of two HN functions to describe the experimental data). The obtained *τ*_*β*_ and *τ*_*γ*_ (*p* = 0.1 MPa) together with *τ*_*α*_ from the previous analysis (Fig. 4a) have been plotted versus *T*_g_/*T*; see Fig. 6. Next, these dependences (i.e., *τ*_*β*_ and *τ*_*γ*_ vs. *T*_g_/*T*) were analyzed using the Arrhenius equation to determine the activation barrier (*E*_*x*_) for the *β* and *γ-*modes:

6 $${\tau }_{x}= {\tau }_{\infty }exp\left(\frac{{E}_{x}}{RT}\right),\quad x = \beta ,\gamma$$

where ${\tau }_{\infty }$ is a pre-exponential factor, and* R* is a gas constant. In Fig. 6, the values of *E*_*β*_ and *E*_*γ*_ estimated with ± 5% uncertainty are presented. As can be observed, *E*_*β*_ increases with increasing compression (*E*_*β*_ = 66, 73, and 81 kJ/mol at 0.1 MPa, 170 MPa and 340 MPa, respectively). Moreover, the *E*_*γ*_ obtained at ambient *p* (= 38 kJ/mol) is clearly lower compared to *E*_*β*_ determined under the same conditions.

From Fig. 6, it is also well seen that *τ*_*β*_ obtained at *p* = 0.1, 170, 340 MPa and rescaled with respect to the *T*_g_, nearly perfectly collapsed onto each other, forming one curve. Such behavior indicates a great sensitivity of the *β*-process to compression. Further confirmation of that is an enormously high value of the activation volume for this relaxation ($\Delta {V}_{\beta }$= 43 cm^3^/mol), estimated from the analysis of isothermal data (dependences of *τ*_*β*_ obtained at 273 K versus *p*; see the upper inset to Fig. 6) using the following formula:

7 $$\Delta {V}_{\beta }=2.303RT{\left(\frac{d {log}_{10}{\tau }_{\beta }}{dp}\right)}_{T}$$

Note that a slightly lower $\Delta V$ for this kind of secondary process has been reported for some other LMW compounds (e.g., posaconazole and itraconazole (23 and 35 cm^3^/mol)^44^, or phenolphthalein dimethyl ether (20–24 cm^3^/mol)^45^), as well as polymers (e.g., polyethylene terephthalate (28–32 cm^3^/mol)^45^, and poly-[(phenyl glycidyl ether)-co-formaldehyde, PPGE (15–19 cm^3^/mol)^45^).

Based on the above facts, including the high value of *E*_*β*_, one can state that the *β*-process in the examined API is a true Johari–Goldstein (JG) relaxation (originating from the local motions of the whole API molecules)^46^. In turn, the faster *γ*-process with clearly lower *E*_*γ*_ has rather an intramolecular character—its source are reorientations of a small part of the molecule.

It is also worth adding that in the case of TICA, the constant ratio *τ*_*α*_ /*τ*_*JG*−*β*_ (data at 0.1 and 170 MPa) for the same *τ*_*α*_ indicates the fulfillment of the isochronal superpositioning of structural (*α*)- and secondary (JG-*β*)-relaxation times—a rule, whose validity has been shown in the literature for many glass-forming systems (including APIs) with different kinds of interactions^40,46–48^.

Finally, we have used the Eyring formula^49^:

8 $$\mathrm{ln}\left({\tau }_{\beta }T\right)=\left[\frac{\Delta {H}_{\beta }}{R}\frac{1}{T}\right]+\left[\mathrm{ln}\left(\frac{h}{{k}_{B}}\right)-\left(\frac{\Delta {S}_{\beta }}{R}\right)\right]$$

where *k*_*B*_ and *h*, are, respectively, the Boltzmann and Planck constants to calculate the entropy and enthalpy of activation for the *β*-relaxation process in TICA ($\Delta {S}_{\beta }$ and $\Delta {H}_{\beta }$, respectively), at ambient and elevated *p.* For this purpose, dependences $\mathrm{ln}\left({\tau }_{\beta }T\right)$ versus 1/*T* (see Fig. S3 in the SI) were fitted to the linear function. Further, we determined $\Delta {S}_{\beta }$ (a measure of the local molecular reorganization induced by the relaxation) from the point of interception with the y axis $\left[\mathrm{ln}\left(\frac{h}{{k}_{B}}\right)-\left(\frac{\Delta {S}_{\beta }}{R}\right)\right]$, while $\Delta {H}_{\beta }$, which is identified with ${E}_{\beta }$ in the Arrhenius equation (Eq. 6), from the slope of the line $\left[\frac{\Delta {H}_{\beta }}{R}\right]$. The obtained values of both parameters are presented versus pressure in the right insets in Fig. 6. As illustrated, both $\Delta {S}_{\beta }$ and $\Delta {H}_{\beta }$ (values comparable to ${E}_{\beta }$ determined at the same thermodynamic conditions) increase with increasing compression. In the context of $\Delta {S}_{\beta }$, the positive value of this parameter means the intermolecular cooperativity of the secondary (*β*)-relaxation in TICA, i.e., the correlation of molecular motions responsible for this process. Importantly, the increase of $\Delta {S}_{\beta }$ with *p* in the case of the examined pharmaceutical is not significant (from 105 J/mol⋅K at 0.1 MPa to 108 J/mol⋅K at 340 MPa). Similar behavior has been observed for some polymers, i.e., PPGE, and poly(vinyl acetate) (PVAc), however, the obtained values of activation entropy were clearly lower (21.3–21.6 J/mol⋅K and 6.2–14.6 J/mol⋅K for the former and latter systems, respectively)^50^. In turn, slightly greater changes of $\Delta {S}_{\beta }$ (from 94 J/mol⋅K at 0.1 MPa to 120 J/mol⋅K at 230 MPa)^44^ have been reported by some of us for the *β*-mode in itraconazole and explained as resulting from some changes in the molecular special arrangement in the compressed sample. Moreover, we have also demonstrated a clear variation in $\Delta {S}_{\beta }$ (from 0.6 J/mol⋅K at 0.1 MPa to 78.1 J/mol⋅K at 115 MPa and 27.8 J/mol⋅K at 160 MPa) for probucol—an API forming weak O–H⋯S bonds at elevated *p* (which are absent at ambient *p*)^51^. In the case of TICA, the slight increase of $\Delta {S}_{\beta }$ with *p* is not associated with the changes in the H-bonding pattern due to compression, as can be deduced from the complementary FTIR studies on the pressure densified glass. One should suppose that it might be related to the solvation of ions (ionic impurities in the sample) around the TICA molecule. Herein, it is worth adding that this problem was well studied in the case of water, where the reorientation dynamics is strongly affected by ions even in strongly diluted solutions^52^. Furthermore, depending on the interaction strength, ions alter or slow down the water dynamics. However, it should be noted that in our system ion concentration was marginal as measured conductivity (${\sigma }_{dc}$) changed with the examined *T* and *p* in the range of 10^−10^–10^−13^ S/cm, while in the vicinity of the *T*_g_, it was of the order of 10^−15^ S/cm; see Fig. S2 in the SI. Moreover, importantly, throughout dielectric measurements, the sample was enclosed in the capacitor, surrounded by an inert media (oil), so the concentration of impurities remained constant. In our experiments, the variation of the dc-conductivity was related only to the mobility changes induced by the viscosity alterations. Based on the above information, it seems that the solvation of ionic impurities has no effect on the value of activation entropy for the *β*-process in TICA. Hence, one can propose that the observed variation of this parameter might result from some small conformational/molecular variations of API molecules at high *p*.

## Conclusions

In this paper, DSC, FTIR, and BDS techniques have been applied to investigate thermal properties, variation in the population of H-bonds, as well as molecular dynamics (in the liquid, supercooled liquid, and glassy states) at ambient and elevated pressure in ticagrelor. Interestingly, our comprehensive HP dielectric investigations demonstrated a strong sensitivity of the structural (*α*)-relaxation to compression (reflected in a high value of d*T*_g_/d*p* = 163 K/GPa parameter) as well as the fulfillment of the TPS rule (a constant shape of the *α*-peak at various combinations of *T* and *p*, for the same *τ*_*α*_). Further FTIR measurements on the ordinary and pressure densified glasses of TICA, as well as a comparison of the obtained data with those determined for another H-bonded API—RTV indicated that the lack of changes in the H-bonding pattern with compression is responsible for that. What is more, additional BDS measurements carried out on the examined substance at *T* < *T*_g_/*p* > *p*_g_ revealed that (1) at isochronal conditions, there is a constant ratio of structural and secondary (*β-*JG-type) relaxation time; (2) the activation entropy for the *β*-mode, Δ*S*_*β*_, has a positive value (which suggests the intermolecular cooperativity of this process) and slightly increases with compression probably due to small conformational/molecular variations of API molecules at high *p*. Our studies open an interesting discussion on the issue concerning the connection between the changes in the H-bonding pattern and the shape of the structural relaxation process at various thermodynamic conditions, which can be probed indirectly by studying the properties of the pressure densified glass.

## Methods

### Materials

Ticagrelor (TICA) [the IUPAC name: (1*S*,2*S*,3*R*,5*S*)-3-[7-[[(1*R*,2*S*)-2-(3,4-difluorophenyl)cyclopropyl]amino]-5-propylsulfanyltriazolo[4,5-d]pyrimidin-3-yl]-5-(2-hydroxyethoxy)cyclopentane-1,2-diol] with molecular weight *M*_w_ = 522.57 g⋅mol^−1^ and purity ≥ 98% was supplied by Sigma Aldrich and used without further purification.

### Preparation of the ordinary and pressure densified glasses of TICA

The ordinary glass (OG) of TICA was obtained by melting the crystalline sample at *T* = 413 K and rapid cooling the obtained liquid (vitrification method). The preparation of the pressure densified glass (PDG) of TICA included, respectively, (1) compressing the sample up to *p* = 400 MPa at *T* = 385 K; (2) cooling it down to* T* = 283 K, and (3) releasing the pressure. Infrared measurements on the OG and PDG (and dielectric studies on the OG) were performed immediately after their preparation.

### DSC

Thermal properties of TICA were examined with the use of a Mettler–Toledo DSC apparatus equipped with a HSS8 ceramic sensor with 120 thermocouples as well as a liquid nitrogen cooling accessory. The instrument was calibrated for temperature and enthalpy using indium and zinc standards while for heat capacity (C_p_)—using a sapphire disc. The crystalline sample of TICA was placed in an aluminum crucible (40 µL) and sealed. Next, it was heated above its melting temperature, quenched (with a rate of 20 K/min), and scanned over a *T* range of 170–430 K. A heating rate was equal to 10 K/min. The melting point was determined as the maximum of the peak, whereas the glass transition temperature as the midpoint of the heat capacity increment.

### BDS

Dielectric measurements of TICA were performed using a dielectric spectrometer (Novo-Control Alpha, Hundsangen, Germany) in the frequency range from 10^−1^ to 10^6^ Hz at atmospheric pressure. The sample was placed between two stainless steel flat electrodes of the capacitor (diameter: 20 mm) with a 0.1 mm gap with a Teflon spacer and mounted on a cryostat. The temperatures were controlled by a Quatro System using a dry nitrogen gas cryostat with stability better than 0.1 K.

For BDS experiments at elevated *p*, a high-pressure Unipress setup (Warszawa, Poland) with a special homemade flat parallel capacitor (diameter: 20 mm; gap: 0.081 mm) was additionally employed. We applied a thin Teflon spacer to maintain a fixed distance between the plates. During the measurement, the sample capacitor was sealed and covered carefully by Teflon tape to separate it from the silicon oil. The pressure was measured using a Nova Swiss tensometric meter with a resolution of 1 MPa, while the temperature was adjusted with a precision of 0.1 K by a refrigerated and heating circulator (HUBER GmbH, Germany).

Comprehensive isothermal and isobaric measurements for the investigated API were performed in the following thermodynamic conditions: in the supercooled liquid state: isothermal measurements: *T* = 347 K (*p* = 0.1–120 MPa), *T* = 381 K (*p* = 120–300 MPa), isobaric measurements: *p* = 0.1 MPa (*T* = 333–363 K), *p* = 120 MPa (*T* = 350–383 K), *p* = 170 MPa (*T* = 357–381 K); in the glassy state: isothermal measurements: *T* = 273 K (*p* = 60–340 MPa), isobaric measurements: *p* = 0.1 MPa (*T* = 173–298 K), *p* = 170 MPa (*T* = 273–333 K), *p* = 340 MPa (*T* = 273–353 K).

## Supplementary Information

Supplementary Information.

## Data Availability

The datasets used and/or analyzed during the current study available from the corresponding author on reasonable request.
