# Supplementary material for: High-pressure studies in the supercooled and glassy state of the strongly associated active pharmaceutical ingredient—ticagrelor
Source: Sci Rep. 2023 Jun 1;13:8890. doi: 10.1038/s41598-023-35772-7 (PMC10235114; doi:10.1038/s41598-023-35772-7)
Supplement: Supplementary file 1 — Supplementary Information. [file 41598_2023_35772_MOESM1_ESM.docx]

**High-pressure studies in the supercooled and glassy state of the strongly associated active pharmaceutical ingredient - ticagrelor**

Paulina Jesionek^1,2,🖂^, Dawid Heczko^3^, Barbara Hachuła^1^, Kamil Kamiński^4^, Ewa Kamińska^2,🖂^

^1^ *Institute of Chemistry, Faculty of Science and Technology, University of Silesia in Katowice, Szkolna 9, 40-007 Katowice, Poland*

^2^ *Department of Pharmacognosy and Phytochemistry, Faculty of Pharmaceutical Sciences in Sosnowiec, Medical University of Silesia in Katowice, Jagiellonska 4, 41-200 Sosnowiec, Poland*

^3^ *Department of Statistics, Department of Instrumental Analysis, Faculty of Pharmaceutical Sciences in Sosnowiec, Medical University of Silesia in Katowice, Ostrogorska 30, 41-200 Sosnowiec, Poland*

^4^ *Institute of Physics, Faculty of Science and Technology, University of Silesia in Katowice, 75 Pulku Piechoty 1, 41-500 Chorzow, Poland*

SUPPLEMENTARY INFORMATION

**BDS DATA**

The data shown in Fig. 4a in the manuscript were analyzed using the Avramov equation^[[1]](#endnote-1)^:

$\tau_{\alpha}=\tau_{\infty}exp\left[ {\ln{(\tau}_{g}/\tau_{\infty})\left( \frac{T_{r}}{T} \right)}^{\alpha_{0}\left( 1-\frac{C}{C_{p_{0}}}\ln\left( 1+\frac{p}{\Pi} \right) \right)}\left( 1+\frac{p}{\Pi} \right)^{\beta} \right]$ , (S1)

where $\tau_{\infty}$ is a relaxation time at extremely high *T*, $\tau_{g}$=$\tau(T_{g})$, $T_{r}$ is a reference temperature lying close to the$T_{g}$, $C_{p_{0}}$ is a specific heat capacity, $C$ is an additional adjustable parameter, $\Pi$ is a constant with the dimension of pressure, $\alpha_{0}$ and $\beta$ are exponential parameters, which are linked to the thermodynamic quantities via the following relations:

$\alpha_{0}=\frac{2C_{p_{0}}}{ZR}$, (S2)

$\beta=\frac{2\alpha_{p}V_{m}}{ZR}\Pi$**,** (S3)

where *Z* represents the degeneracy of the system,$\alpha_{p}$ is a volume expansion coefficient at ambient *p*, and $V_{m}$ is a molar volume. The parameters of equation S1 determined from the global numerical fitting (Fig. 4a) are presented in Table S1.

The separate isobars and isotherms together with the Avramov fitting curves are shown in Fig. S1.

**Figure S1.** Temperature (a) and pressure (b) dependence of structural relaxation times obtained for TICA from isobaric and isothermal dielectric measurements. Black lines represent fits using the modified Avramov function.

**Table S1.** Parameters of the modified Avramov equation (equation S1) obtained from the analysis of *τ_α_* (*T*,*p*) dependencies for TICA.

|  | **TICA** |
| --- | --- |
| log_10_(*τ_∞_*[s]) | -8.19±0.17 |
| *T_r_* [K] | 319.0±0.20 |
| *α*_0_ | 10.23±0.37 |
| *C/C_p_*_0_ | 0.27±0.009 |
| Π [MPa] | 217.96±13.05 |
| *β* | 1.189±0.068 |
| Adj. R-Square | 0.999 |

**Figure S2.** The logarithm of dc conductivity plotted versus the logarithm of structural relaxation times determined from the analysis of isobaric (a) and isothermal (b) dielectric data.

**Figure S3.** The dependences $\ln\left( \tau_{\beta}T \right)$ vs. 1/*T* obtained for TICA at *p* = 0.1, 170 and 340 MPa. Solid lines represent linear fits.

**References**

1. . Avramov, I. Pressure dependence of viscosity of glass-forming melts. *J. Non-Cryst. Solids* **262**, 258–262 (2000). [↑](#endnote-ref-1)
